# Supplementary figures and images for: Mimicking seasonal changes in light-dark cycle and ambient temperature modulates gut microbiome in mice under the same dietary regimen
Source: PLoS One. 2023 Feb 15;18(2):e0278013. doi: 10.1371/journal.pone.0278013 (PMC9931110; doi:10.1371/journal.pone.0278013)

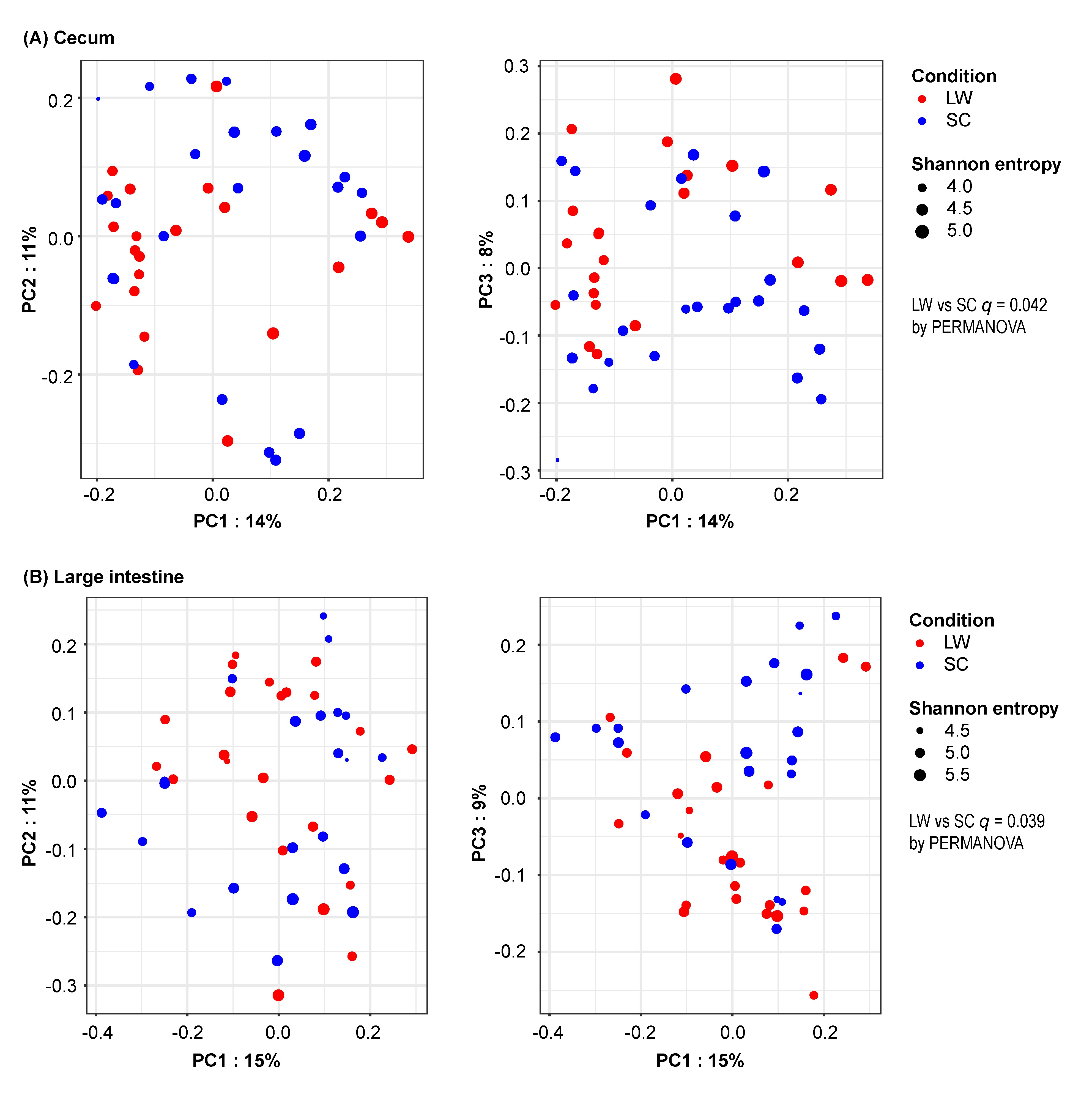

Supplement: S1 Fig — Principal coordinate analysis (PCoA) plot with Bray Curtis distance in (A) the cecum and (B) the large intestine microbiome of CBA/N mice. Each point represents one sample, and the color of the dots indicates the seasonal conditions (LW, SC). The number of individuals at each sampling time was 4. However, the number was 2 at two points (cecum LW 8:00 and large intestine SC 16:00) because some samples failed in the quality control process. (TIF) [file pone.0278013.s001.tif]

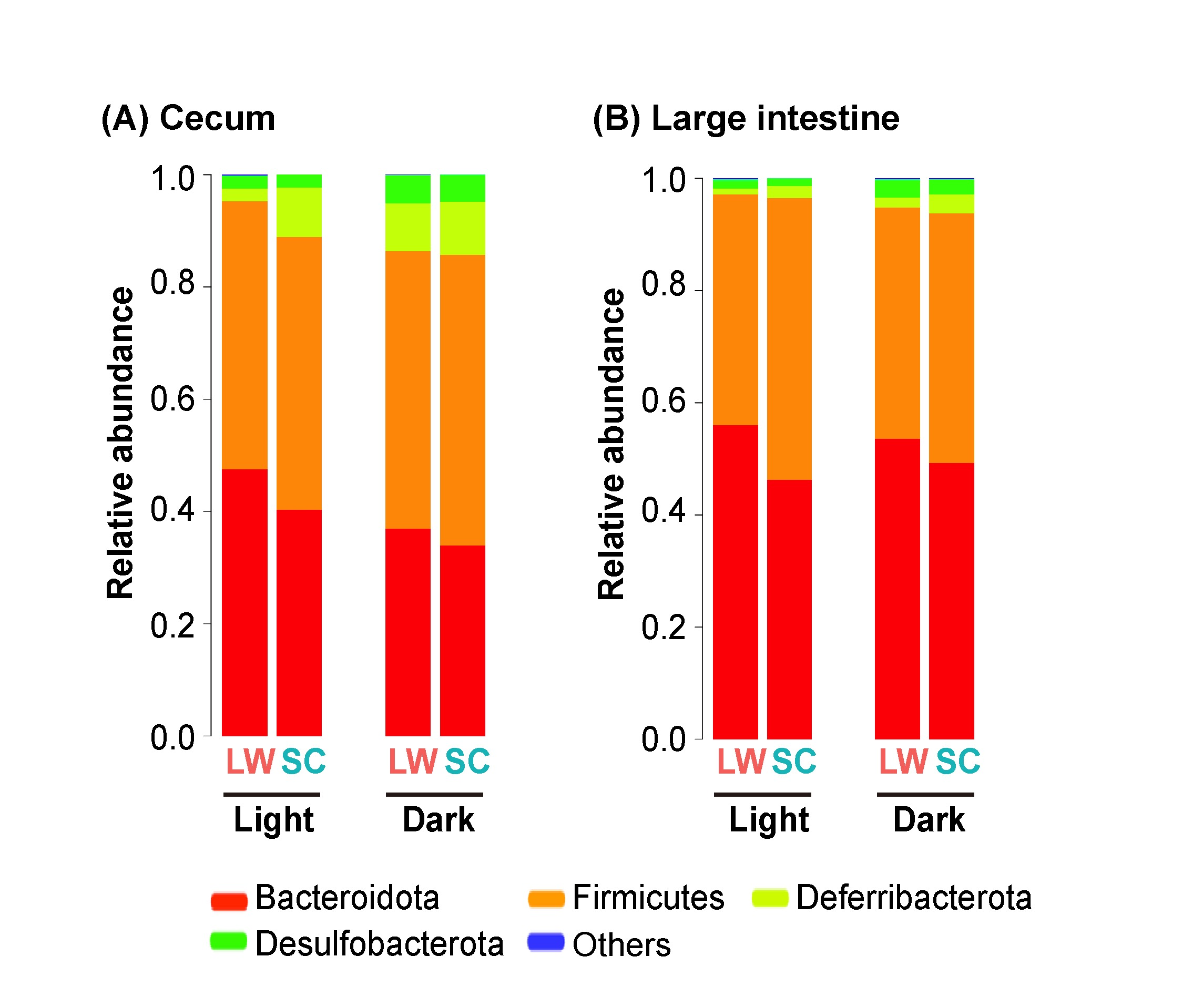

Supplement: S2 Fig — Relative abundance at phylum level in (A) the cecum and (B) the large intestine of CBA/N mice at day and night. Day is the data from the middle of the day (12:00), while night is the data from the middle of the night (0:00). The number of individuals at each bar is 4. (TIF) [file pone.0278013.s002.tif]

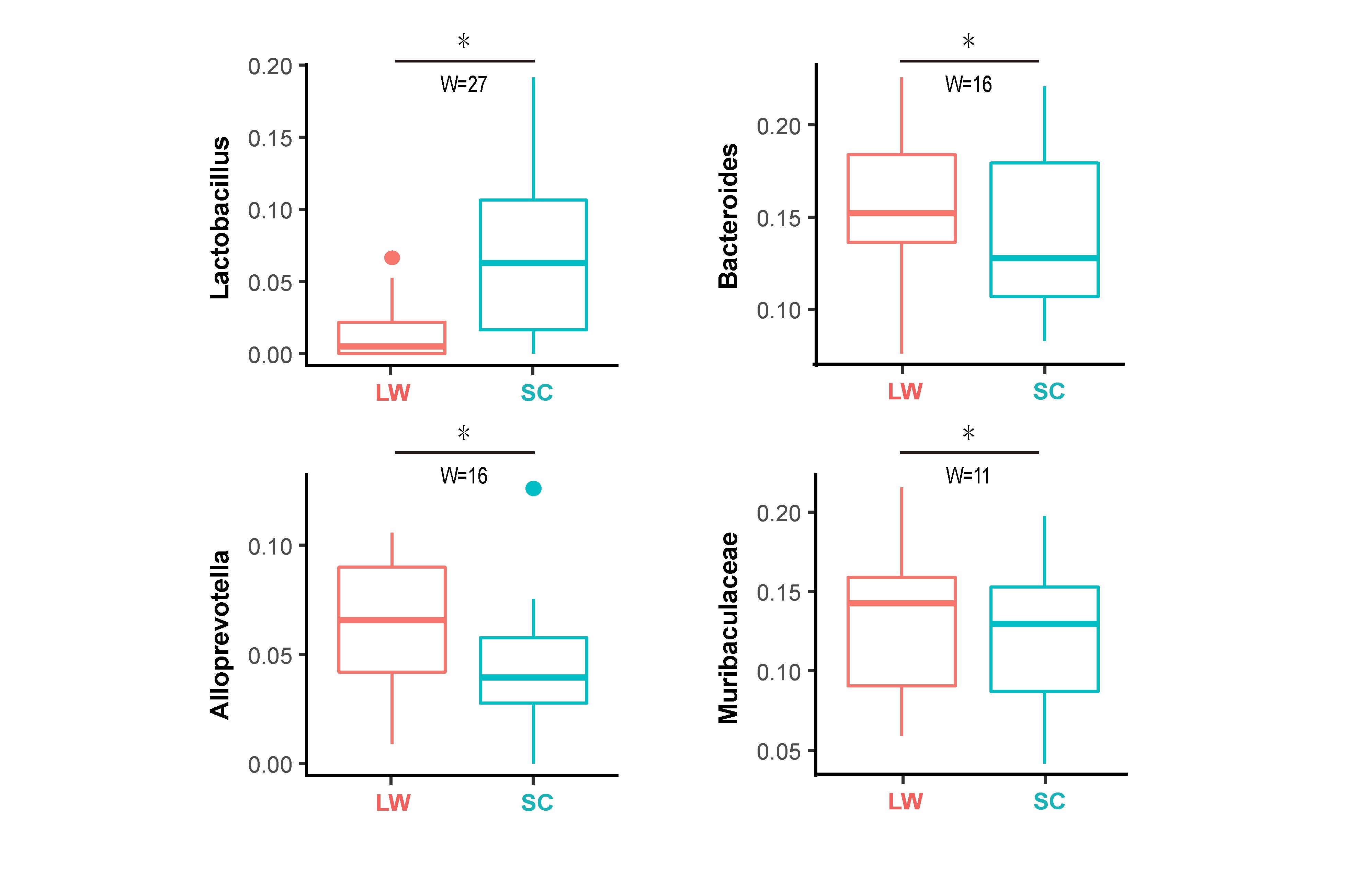

Supplement: S3 Fig — Analysis of composition of microbiomes (ANCOM) detected the significant differences between LW and SC in Lactobacillus, Bacteroides, Alloprevotella and Muribaculaceae. W represents the number of null hypotheses rejected when statistics are performed to see if there is a difference between samples for a particular bacterium. The number of LW individuals is 22, and SC individuals is 24. (TIF) [file pone.0278013.s003.tif]
